# Infection with gut parasites correlates with gut microbiome diversity across human populations in Africa

**DOI:** 10.1080/19490976.2025.2587966

**Published:** 2025-12-08

**Authors:** Mirabeau M. Ngwese, Bayode R. Adegbite, Jeannot F. Zinsou, J. Liam Fitzstevens, Victor T. Schmidt, Paul Alvyn N. Moure, Moustapha N. Maloum, Alexander V. Tyakht, Kelsey E. Huus, Nicholas D. Youngblut, Peter G. Kremsner, Ayola A. Adegnika, Ruth E. Ley

**Affiliations:** a Department of Microbiome Science, Max Planck Institute for Biology, Tübingen, Germany; b Centre de Recherches Médicales de Lambaréné (CERMEL), Lambaréné, Gabon; c Institut für Tropenmedizin, Eberhard-Karls-Universität Tübingen, Germany; d German Center for Infection Research (DZIF), Tübingen, Germany; e Cluster of Excellence EXC 2124 Controlling Microbes to Fight Infections, University of Tübingen, Germany

**Keywords:** Soil-transmitted helminths, gut microbiome, qPCR, sequencing, alpha diversity, beta diversity, host-microbiome interactions, microbiome-parasite associations

## Abstract

Soil-transmitted helminths (STH) are common in (sub)tropical regions and primarily affect impoverished populations. These parasites reside in the gut, where they interact with both the microbiota and host immunity. Clinical STH detection is laborious and often not performed within the context of gut microbiome studies. Here, we present a proof-of-concept study assessing whether fecal metagenome data could be used to assess STH infection, and to relate STH infection to microbiome features. We leveraged 310 gut metagenomes obtained from mother-child pairs in two different locations in Gabon: one rural and one semi-urban, and assessed the presence of four STH species (*Ascaris lumbricoides*, *Strongyloides stercoralis*, *Trichuris trichiura*, and *Necator americanus*) using qPCR. Sequence data were used to characterize the microbiomes and to detect these parasites. Metagenomic read mapping and genome coverage metrics closely matched qPCR detection patterns. Within-location analyses revealed that parasite species richness was associated with microbiome diversity and taxonomic composition, with the strongest associations observed in children from the rural site. Applying this approach to published data from five additional African cohorts identified context-specific parasite-microbiome associations, as well as a modest but reproducible association between microbiome alpha diversity and parasite infection. These findings highlight the potential of shotgun metagenomics for concurrent parasite detection and microbiome profiling across diverse geographic and demographic contexts.

## Introduction

Soil-transmitted helminth infections are endemic in developing countries, with over 1.5 billion people - representing 24% of the global population - affected by soil-transmitted helminths (STH).^1^ These infections are predominantly found in tropical and subtropical regions, particularly sub-Saharan Africa.^2^ ^,^ ^3^ Transmission occurs through oral-faecal contact via contaminated soil and transcutaneously through larval penetration of the skin. Infections can spread within and between communities through shared environments. The prevalence of STHs in rural communities is often poorly documented, especially in countries with limited resources and lacking mass drug administration (MDA) programs. MDA strategies, including deworming of high-risk populations and the widespread administration of anthelmintic drugs, are essential for controlling and reducing the morbidity associated with worm infections.^4^

The World Health Organisation (WHO) classifies the STH species *Ascaris lumbricoides*, *Trichuris trichiura*, Hookworms (*Necator americanus* and *Ancylostoma duodenale)* and *Strongyloides stercoralis* as neglected tropical diseases. These first three STHs contributed to an estimated 1.9 million disability-adjusted life years worldwide in 2019.^5-7^ Strongyloidiasis, caused by *S. stercoralis*, further affects an estimated 30 to 100 million people worldwide.^8^ At-risk groups include young children, preschool and school-age children, adolescent girls, women of reproductive age, pregnant women and adults with certain high-risk jobs such as tea-pickers or miners.^5^ ^,^ ^9^ STH parasites often cause significant nutritional deficiencies, affecting growth and cognitive development in children.^10^ They can also impact the behaviour of the patient, as STH infections have been associated with reduced attention, memory, and learning abilities in children,^10^ ^,^ ^11^and experimental infection models have demonstrated impairments in spatial memory.^12^ In addition, they can lead to intestinal inflammation and malabsorption.^13-15^

STHs are members of the gut microbiome. As such, they interact with other components of the microbiome such as Bacteria and Archaea, and these interactions can impact the success of parasite colonisation and the outcome of parasitic infections.^16^ Conversely, parasite colonisation can affect the host's interaction with the microbiome.^17-19^ When parasites colonise the gut, changes occur in the gut barrier, such as the epithelial mucus layers, which can subsequently alter the composition of microbiota.^20^ ^,^ ^21^ Helminths can influence host-microbiome interactions by modulating immune signalling pathways and altering nutrient availability, thereby creating conditions that support their persistence within the gut ecosystem.^22^ Furthermore, helminth-induced immunomodulation fosters a tolerogenic environment, which may encourage the growth of specific microbial taxa, indicating a potential mutualistic relationship between helminths and certain microbial communities.^23^ Therefore, interactions between parasites and microbes may play significant roles in modifying host physiology and influencing disease outcomes.^24^

The investigation of parasite-microbiome relationships across diverse populations has been limited in part because quantifying STH load is laborious and time-intensive and is not always done in conjunction with microbiome studies. Recently, a number of groups have reported the possibility of detecting parasite presence by metagenomic sequencing in livestock stool^25^ ^,^ ^26^ and the detection of unicellular parasites, such as Blastocystis, in human faecal metagenomes.^27^ Although metagenomic sequencing shows promise for detecting a wide range of pathogens including STH, its diagnostic performance has not yet been fully validated against traditional diagnostic methods such as microscopy and PCR,^28^ highlighting the need for further standardisation and targeted validation studies.

Here, we examined STH load in subjects sampled in Gabon using qPCR to compare with estimates generated from gut metagenomes. We then examined the relationship between STH infection with gut microbiome diversity from the same individuals. Subjects consisted of mother-child pairs from two different communities: the semi-urban region of Lambaréné, where parasite prevalence is well-documented,^29^ ^,^ ^30^ and the rural "Ikobey" region, which lacks parasite distribution data. We then expanded our analysis by leveraging publicly available metagenomic data from five additional studies in diverse African populations. Our results indicate that metagenome data can be used to assess STH richness, revealing reproducible patterns in microbiome-parasite associations across populations.

## Results

### Prevalence of parasites in samples from Gabon

**Figure 1.**
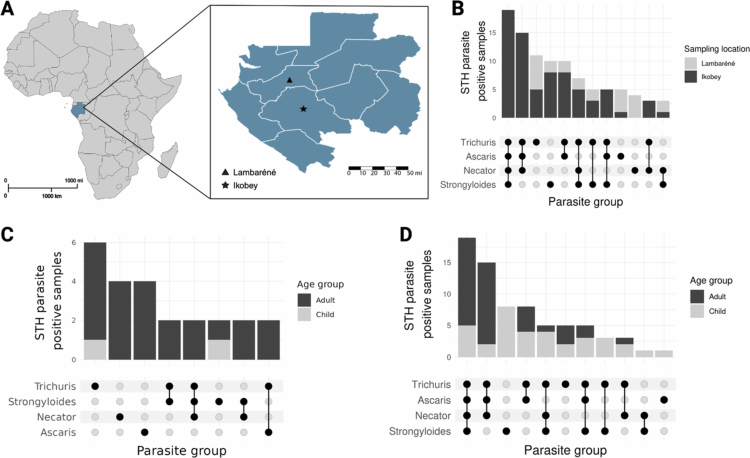
Sampling sites and prevalence of four STH species in Lambaréné and Ikobey. A) Map of Gabon showing the sampling locations (★ = Ikobey villages, ▲ = Lambaréné). **B)** Total number of STH-positive samples based on combined microscopy and qPCR data, displayed by sampling location. **C)** Total number of STH-positive samples by qPCR data, displayed by age group (Lambaréné). **D)** Total number of STH-positive samples from Ikobey, determined by qPCR data.

We collected 310 human stool samples from mothers and their children in Gabon (Figure 1A) as described in.^31^ This dataset comprises matched mother-child pairs from two distinct regions, the rural Ikobey (*n* = 40 pairs) and the semi-urban Lambaréné (*n* = 115 pairs). While the original study^31^ was designed to investigate microbial strain transmission and cophylogeny, we leveraged this well-characterised cohort to address a complementary research question focused on parasite-microbiome associations. A total of 230 samples from Lambaréné and 80 from Ikobey were shipped to Germany for metagenomic sequencing and for qPCR-based detection of STHs. 259 were previously reported^31^ and 51 were newly generated for this study. The average age of adults was 27 ± 6.7 (s.d.) years (Lambaréné = 26.3, Ikobey = 30.7), while that of children was 40 ± 45 (s.d.) weeks (Lambaréné = approximately 26 weeks [0.5 years], Ikobey = approximately 73 weeks [1.4 years]). Adults and children tended on average to be older in Ikobey compared to Lambaréné (Wilcoxon rank sum test, *p* = 6e−04 and *p* = 0.0032, respectively; Table 1).

**Table 1.** Age distribution and proportions of infection with one or more STH parasites (by qPCR: ct<36) between groups in the Lambaréné and Ikobey sample populations.

	Sampling site				
Category	Lambaréné		Ikobey		*p*-values
Mean age (years)	mothers: 26.3		mothers: 30.7		*p* = 6e^−04^
	Children: 0.5		Children: 1.4		*p* = 0.0032
Proportion of infection *n* (%)	Mothers: 22 (19.1)		Mothers: 38 (95)		
	Children: 2 (1.9)		Children: 35 (87.5)		
Multiparasitism	Mothers (*n* = 115) Single infections (14)		Mothers (*n* = 40) Single infections (3)		
	Children (*n* = 115) Single infections (2)		Children (*n* = 40) Single infections (11)		
	Mothers: Double infections (7)		Mothers: Double infections (5)		
	Children: Double infections (0)		Children: Double infections (11)		
	Mothers: Triple infections (1)		Mothers: Triple infections (16)		
	Children: Triple infections (0)		Children: Triple infections (8)		
	Mothers: Quadruple infections (0)		Mothers: Quadruple infections (14)		
	Children: Quadruple infections (0)		Children: Quadruple infections (5)		

Individual parasite prevalence (%)	Location			
	Adults	Children	Adults	Children
***A. lumbricoides***	5.2	0.0	82.5	37.5
***T. trichiura***	9.6	0.9	95.0	60.0
***N. americanus***	6.1	0.0	72.5	35.0
***S. stercoralis***	6.1	0.9	42.5	60.0

***A. lumbricoides = Ascaris Lumbricoides, T. trichiura = Trichuris trichiura.*** ***N. americanus = Necator americanus, S. stercoralis = Strongyloides. stercoralis.***

We focused on the four STH species previously reported as prevalent in this population *(i.e.*, *A. lumbricoides*, *T. trichiura*, *N. americanus* and *S. stercoralis*).^32^ ^,^ ^33^ To characterise the distribution of intestinal parasites in this cohort, we first examined parasite counts per individual across age groups within each location. Linear models revealed significant differences by age in both settings. In both semi-urban Lambaréné and rural Ikobey, children had significantly lower parasite counts than adults (Linear model: *p* = 2.3 × 10^−^⁵ and *p* = 2.8 × 10^−^⁴; respectively; Table S1a). Post-hoc Tukey multiple comparisons confirmed these differences after false discovery rate (FDR) correction (Lambaréné: FDR-adjusted *p* = 2.3 × 10^−^⁵; Ikobey: FDR-adjusted *p* = 2.8 × 10^−^⁴; Table S1b), indicating that adults generally harboured more parasites per individual than children (Figure 1B,C). These findings establish the baseline patterns of parasite burden necessary for subsequent analyses of infection risk and prevalence.

To assess whether age influenced the likelihood of being infected, we applied logistic regression models to parasite presence/absence data. In Lambaréné, children had significantly lower odds of infection than adults (odds ratio = 0.075, *p* = 5.6 × 10^−^⁴). In Ikobey, the same trend was observed, but it did not reach statistical significance (odds ratio = 0.37, *p* = 0.25; Table S1c), reflecting the overall high prevalence of infection in this rural community. These models indicate that age is an important determinant of infection in the semi-urban population, whereas in high-prevalence rural settings, age-related differences are less pronounced.

To quantify infection prevalence and evaluate age-related differences within each location, we performed chi-squared tests for each parasite species. In Lambaréné, adults were more likely than children to be infected with *A. lumbricoides* (*p* = 0.0515), *T. trichiura* (*p* = 0.0152), and *N. americanus* (*p* = 0.0340), while *S. stercoralis* prevalence did not differ significantly by age (*p* = 0.0822). Similarly, in Ikobey, significant age differences were observed for *A. lumbricoides* (*p* = 0.000837), *T. trichiura* (*p* = 0.002), and *N. americanus* (*p* = 0.00452), but not for *S. stercoralis* (*p* = 0.18). All *p*-values were FDR-adjusted for multiple comparisons. These results confirm that age-related differences in parasite infection are evident for most species, though the effect is more pronounced in the semi-urban Lambaréné population.

To evaluate whether STH infection status was more similar within mother–child pairs than expected by chance, we performed a permutation-based concordance analysis stratified by location and parasite species. Across all species and locations, observed concordance did not differ from the null distribution generated by randomising child infection status within locations (all *P* > 0.05; results not shown). These findings suggest no evidence that mothers with STH infection were more likely to have children infected with the same parasites, or vice versa.

Finally, to summarise overall parasite prevalence, we examined age-stratified infection rates for each species in both locations. In Ikobey, prevalence in adults versus children was 82.5% versus 37.5% for *A. lumbricoides*, 72.5% versus 35% for *N. americanus*, 42.5% versus 60% for *S. stercoralis*, and 95% versus 60% for *T. trichiura*. In Lambaréné, prevalence was substantially lower, with adults showing 5.2% versus 0% for *A. lumbricoides*, 6.1% versus 0% for *N. americanus*, 6.1% versus 0.9% for *S. stercoralis*, and 9.6% versus 0.9% for *T. trichiura* (Table 1). These findings highlight both the higher parasite burden in the rural Ikobey region and the marked age-related differences in infection risk within the semi-urban population. Together, these results provide a view of how parasite prevalence and burden vary with both geography and age, establishing a foundation for subsequent analyses of parasite–microbiome associations.

### Associations of gut microbiome alpha and beta diversity with the number of STH species

We next sought to investigate associations between the microbiome and parasite burden (richness) estimated by qPCR (*n* = 310). To evaluate gut microbiome diversity at the species level, we employed metagenomic profiling against a custom database created from the Genome Taxonomy Database (GTDB Release 202).^34^ This approach significantly improved the classification of reads compared to the standard Kraken database, increasing the percentage of classified reads from 38.0% ± 26.4 (s.d.) to 81.8% ± 5.6 (s.d.).

**Figure 2.**
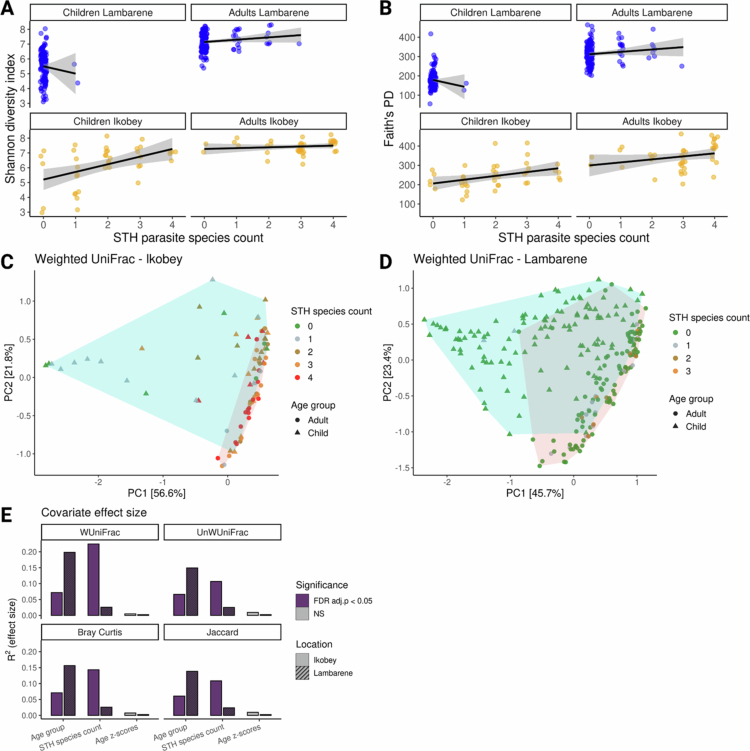
Gut Microbiome Diversity in Relation to STH species count, Age Group, and Location. A) Correlation between gut microbiome Shannon diversity index and STH species count for adults and children by location (correlation method: LM). For children, *R* ^*2*^ = 0.23 in Ikobey and *R* ^*2*^ = 0 in Lambaréné; for adults, *R* ^*2*^ = 0.02 in both Ikobey and Lambaréné. **B)** Correlation between Faith’s Phylogenetic Diversity (PD) and STH species count for adults and children by location (correlation method: LM). For children, *R* ^*2*^ = 0.15 in Ikobey and *R* ^*2*^ = 0.01 in Lambaréné; for adults, *R* ^*2*^ = 0.07 in Ikobey and *R* ^*2*^ = 0.02 in Lambaréné. **C)** Principal Coordinates Analysis (PCoA) of weighted UniFrac values for Ikobey samples (*n* = 80), coloured by STH species count and shaped by age group (Adults = circles, children = triangles). **D)** PCoA of weighted UniFrac values for Lambaréné samples (*n* = 230), coloured by STH species count and shaped by age group (Adults = circles, children = triangles). **E)** Effect size (R²) of each covariate plotted against significance (FDR-adjusted *p* < 0.05 = *, ns = non-significant) for all samples in both locations. Effect sizes were calculated using four beta diversity metrics: Bray-Curtis, Jaccard, Weighted UniFrac, and Unweighted UniFrac. Abbreviations: WUniFrac = Weighted UniFrac, UnWUniFrac = Unweighted UniFrac.

To assess within-sample (alpha) diversity, and better account for the impact of age and parasite richness, we modelled Shannon entropy and Faith’s phylogenetic diversity (PD), using linear models stratified by sampling location and age group (*n* = 307 samples with age data). These models included parasite species richness (parasite count) and standardised age (z-scores, Figure S1A &B) as predictors (Table S2a-b). Among children in Ikobey, parasite richness was significantly and positively associated with Shannon diversity (estimate = 0.33, FDR-adjusted *p* = 0,030). Age was also strongly associated with both Shannon diversity and Faith’s PD in this group (FDR-adjusted *p* = 1.44 × 10^−3^ and 4.1 × 10^−5^, respectively). In contrast, no significant association with parasite richness was observed in Ikobey adults or in either age group from Lambaréné, although weak trends were noted in adults (Figure 2A,B; Table S2a-b). Faith’s PD was not associated with parasite richness in any group, but remained positively correlated with age in children from both locations (Ikobey: FDR-adjusted *p* = 4.1 × 10^−5^; Lambaréné: FDR-adjusted *p* = 6.4 × 10^−3^), but not in adults (Table S2a-b). These findings suggest that both parasite diversity and bacterial diversity increase with age in children, particularly those in Ikobey, but that there is little relationship between microbiome diversity and parasite burden in Gabonese adults.

We then assessed between-sample (beta) diversity using four distance metrics: Bray-Curtis, Jaccard, weighted UniFrac (Figure 2C,D), and unweighted UniFrac (Figure S1C&D), followed by PERMANOVA models stratified by sampling location (Lambaréné and Ikobey). In Lambaréné, age group explained the largest share of variance in microbiome composition across all metrics (*R²* = 0.138 to 0.198), while parasite richness accounted for substantially less (*R²* = 0.024 to 0,027). In Ikobey, the pattern was reversed: parasite richness explained the most variation across all four metrics (*R²* = 0.107 to 0.225), exceeding the contribution of the age group (*R²* = 0.061 to 0.073) (Table S2c; Figure 2E).

These findings show that parasite richness contributes to variation in microbiome composition, particularly in Ikobey children where overall parasite prevalence is higher. In contrast, in the more urban setting of Lambaréné, age group is the dominant structuring factor. Together, these analyses reveal that both age and parasite burden can contribute to microbiome diversity in children, but their relative role depends on ecological context and exposure.

To assess these patterns in the pooled dataset while explicitly accounting for geography (sampling location), we ran PERMANOVA models including location as a covariate. Parasite burden remained significantly associated with beta diversity across all metrics (*R²* = 0.047−0.063, FDR-adjusted p ≤ 0.0015). Age explained the largest share of variance (*R²* = 0.101–0.152), while location contributed comparatively little (*R*² = 0.005–0.008) but reached significance for unweighted UniFrac, Bray–Curtis, and Jaccard (FDR-adjusted *p* = 0.006–0.015). Using age group instead of continuous age yielded consistent results and slightly higher explanatory values, while avoiding sample loss from missing age data. These pooled analyses reinforce that age and parasite burden are stronger drivers of microbial community structure than geography in this cohort.

### Specific microbial taxa are differentially abundant by STH species richness

**Figure 3.**
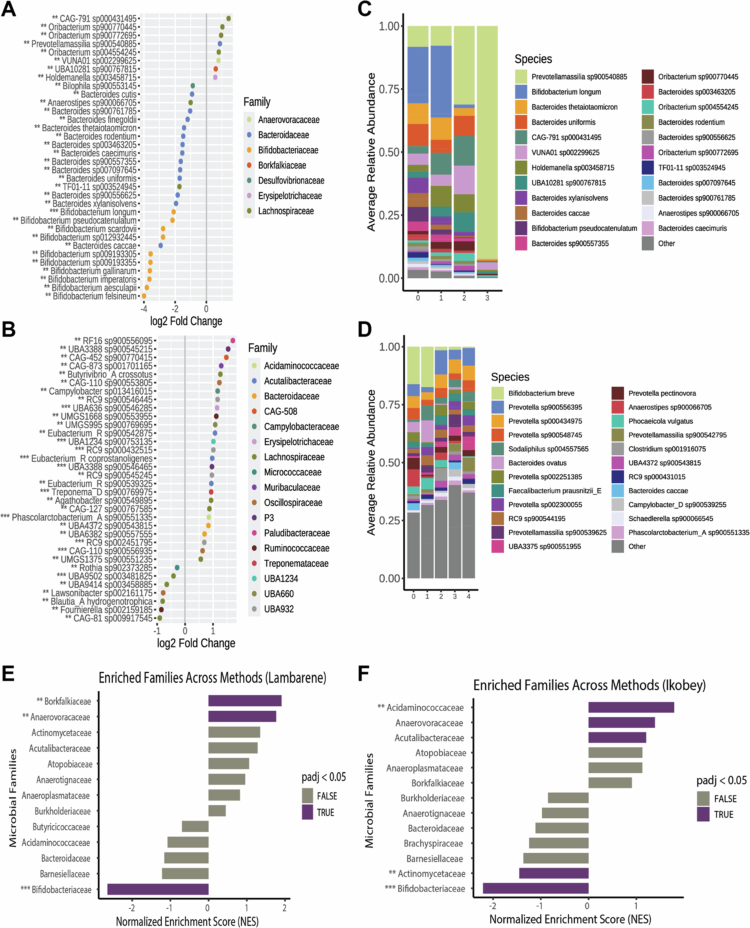
Differentially abundant microbial species and enriched bacterial families by STH parasite species richness across sampling locations. A-B) Log2 fold changes in microbial species abundance associated with STH parasite count, separately for Lambaréné (A), and Ikobey (B), identified using DESeq2, MaAslin2, and ANCOM-BC, while adjusting for age group (Adult vs. Child). Shown are the top 30 species found significant (FDR-adjusted *p* < 0.05) in at least two of the three methods. **C-D)** Relative abundances of the top all differentially abundant species in Lambaréné (C), and Ikobey (D), based on overlap across at least two methods. The top 23 species are coloured by bacterial family; remaining species are grouped as “Other” **E-F)** Normalised enrichment scores of bacterial families significantly overrepresented in the differentially abundant species in Lambaréné (E), and Ikobey (F), based on fast gene set enrichment analysis (FGSEA). Species-level abundances were pre-filtered to retain only those present in>50% of samples from both locations. The log2 fold changes are shown from the method reporting the highest effect size per species. Species identified by all three methods are annotated with ***, and those by two methods with **).

Having observed significant differences in microbiome diversity by parasite richness, we next sought to identify specific microbial taxa associated with the number of STH species. To account for substantial geographic variation in microbiome composition, we conducted separate differential abundance analyses for each sampling location (Lambaréné and Ikobey), while accounting for age group. We applied three complementary methods, DESeq2, MaAsLin2, and ANCOM-BC, each with distinct normalisation strategies, to ensure robustness.^35-37^ Because individual differential-abundance tools can yield variable results and differ in their susceptibility to false positives, we focused on taxa consistently identified across methods, thereby emphasising signals most likely to represent biologically robust associations rather than method-specific artifacts. In Lambaréné, DESeq2 identified 108 species significantly associated with parasite richness, while MaAsLin2 and ANCOM-BC identified 29 and 37 species, respectively (FDR-adjusted *p* < 0.05), yielding a combined total of 174 unique species. Of these, 34 (19.5%) were detected by at least two methods (Table S3a), while 140 were method-specific. In Ikobey, DESeq2, MaAsLin2, and ANCOM-BC identified 252, 633, and 127 significant species, respectively, with 182 (18%) overlapping species and 830 method-specific (Table S3b). The top 30 overlapping species per location are shown in Figure 3A,B. Relative abundance profiles for overlapping species are visualised in Figure 3C,D, displaying the 23 most abundant species individually and grouping the remainder as “Other.” Proportions are scaled within the subset and do not reflect total community composition. Ten consistently significant species were shared across both locations, primarily from Bacteroidaceae (*n* = 4), Lachnospiraceae (*n* = 5), and Anaerovoracaceae (*n* = 1).

Focusing on species identified by at least two methods, we observed contrasting patterns of association across locations. In Lambaréné, most overlapping species were negatively associated with parasite richness, particularly within Bacteroidaceae (*n* = 13), Bifidobacteriaceae (*n* = 10), and Lachnospiraceae (*n* = 2). Fewer species were positively associated, including members of Lachnospiraceae (*n* = 4) and one species each from Bacteroidaceae, Anaerovoracaceae, Borkfalkiaceae, and Erysipelotrichaceae (Figure 3A, Table S3a). In contrast, Ikobey exhibited a dominance of positive associations, particularly from Lachnospiraceae (*n* = 23), Bacteroidaceae (*n* = 20), UBA932 (*n* = 14), and Oscillospiraceae (*n* = 12), as well as other families including Acutalibacteraceae, UBA660, Ruminococcaceae, Streptococcaceae, Muribaculaceae, Campilobacteraceae, and Erysipelotrichaceae (3–8 species each). Negative associations were found mainly in Bacteroidaceae (*n* = 13), Lachnospiraceae (*n* = 9), and Coriobacteraceae (*n* = 9), with smaller contributions from Bifidobacteriaceae (*n* = 2) and others (Figure 3B, Table S3b).

To identify taxonomic families enriched among differentially abundant species, we applied fast gene set enrichment analysis (FGSEA) using ranked association statistics.^38^ In Lambaréné, 16 families were significantly enriched (padj<0.05), primarily from the classes Clostridia (*n* = 6), Bacteroidia (*n* = 3), Bacilli (*n* = 2), and Gammaproteobacteria (*n* = 2), with one family each from Actinomycetia, Coriobacteriia, and Negativicutes (Figure 3E, Table S3c). Most of these families had positive normalised enrichment scores (NES), suggesting that their constituent species tended to be positively associated with parasite richness. A smaller number showed negative NES values, indicating the opposite trend. In Ikobey, 26 families were enriched (FDR-adjusted *p* < 0.05), most with positive NES values, including members of Clostridia (*n* = 6), Bacilli (*n* = 4), Gammaproteobacteria (*n* = 2), and Negativicutes (*n* = 2), with additional contributions from several other classes (Figure 3F, Table S3d). Families with negative NES values included Actinomycetia (*n* = 2), Negativicutes (*n* = 2), and one family each from Clostridia, Bacilli, Gammaproteobacteria, Bacteroidia, and Coriobacteriia.

Across both sites, 12 families were significantly enriched by FGSEA. Of these, nine families—Anaerovoracaceae, CAG−272, Oscillospiraceae, Erysipelotrichaceae, Streptococcaceae, UBA932, Veillonellaceae, Lachnospiraceae, and Pasteurellaceae—were generally associated with higher parasite richness, with most constituent species showing positive associations in at least one dataset. For instance, Anaerovoracaceae and Erysipelotrichaceae were positively associated in both locations, whereas Pasteurellaceae, Veillonellaceae, CAG−272, and UBA932 showed predominantly positive associations in Ikobey but not in Lambaréné. Bifidobacteriaceae, by contrast, exhibited only negative associations at both sites. Families such as Lachnospiraceae, Oscillospiraceae, and Streptococcaceae displayed mixed patterns, with more positive than negative associations across both locations. For example, Lachnospiraceae included 4 positively and 2 negatively associated species in Lambaréné, and 23 positives vs. 9 negatives in Ikobey.

These results suggest that parasite–microbiota associations vary by geographic location, taxonomic composition, and direction of association. The recurrent enrichment of certain families across sites may reflect shared microbial responses to STH exposure, although the specific functional contributions of these taxa remain to be elucidated.

### Functional pathway shifts in relation to parasite burden

Given that microbial taxa differing by parasite burden often cluster within functionally coherent groups, we investigated whether gut microbial metabolic pathways also differed between parasite-positive and parasite-negative individuals. We applied the same three statistical frameworks used for taxonomic differential abundance (DESeq2, MaAsLin2, ANCOM-BC) to HUMAnN3-derived unnormalized, unstratified pathway abundance profiles, stratified by sampling location (Lambaréné, Ikobey) and adjusting for age group. Analyses were performed with both continuous age and age group; using age group retained all pathways identified with continuous age and additionally detected more, as it reduced sample loss from missing age values and increased statistical power in the stratified analyses. For pathway analyses, significance was defined as FDR-adjusted *p* < 0.1, and only pathways detected by at least two methods were considered robust. In Lambaréné, DESeq2 identified one differentially abundant pathway (PWY−622: starch biosynthesis), MaAsLin2 identified four (PWY−6641: superpathway of sulfolactate degradation, PWY−6148: tetrahydromethanopterin biosynthesis, PWY−6595: superpathway of guanosine nucleotides degradation plants, PWY−6478: GDP-D-glycero-*α*-D-manno-heptose biosynthesis), and ANCOM-BC identified none. No pathway overlapped between the three methods (Table S3e). Of the MaAsLin2 results, PWY−664: superpathway of sulfolactate degradation and PWY−6148: tetrahydromethanopterin biosynthesis were enriched in parasite-positive individuals (positive log₂ fold change), while the others, including PWY−622: starch biosynthesis, were depleted.

In Ikobey, where parasite prevalence was higher, DESeq2 identified 25 pathways, MaAsLin2 identified 76, and ANCOM-BC identified 35, with 27 pathways overlapping between all three methods. Of these, only PWY66−409: superpathway of purine nucleotide salvage and PWY−7323: superpathway of GDP-mannose derived O-antigen building blocks biosynthesis were enriched, while the remaining 25 were depleted (Figure S2; Table S3f). No differentially abundant pathways overlapped between locations, although several amino acid biosynthesis pathways that were significantly depleted with STH in Ikobey showed a similar, non-significant trend to depletion in Lambaréné.

Overall, functional pathway differences were minimal in Lambaréné but pronounced in Ikobey, echoing patterns observed in the taxonomic profiles and aligning with the higher parasite prevalence in Ikobey. These findings suggest that helminth-associated microbiome changes in high-prevalence settings extend beyond taxonomic shifts to include broad alterations in predicted metabolic capacity.

### Quantification of parasites by qPCR correlates with metagenome sequence data

Next, we explored the feasibility of detecting and quantifying STH parasites directly from shotgun metagenome data. After filtering out bacterial and archaeal sequences, we employed KrakenUniq^39^ to classify the remaining sequences against a custom database of the four target STH species, obtaining k-mer counts for each reference genome. To complement the k-mer based abundance estimates, genome coverage was assessed for the same species (Table S10) by mapping Kraken2-unclassified reads from a subset of 30 Gabonese samples to the corresponding reference genomes. The 30 samples were selected to include individuals with the highest k-mer counts per species, spanning both adults and children.

Mapped contigs ranged in length from 134 bp (*N. americanus*) to 29.16 Mb (*T. trichiura*), with median coverage values varying substantially between species (Figure S3A). Minimum average per-contig coverage ranged from 0.000077x (*T. trichiura*) to 0.00244x (*S. stercoralis*), while maximum values ranged from 0.0386x (*T. trichiura*) to 632x (*A. lumbricoides*). For all four species, high-coverage contigs tended to be shorter fragments, whereas long contigs generally showed lower average coverage. This pattern is consistent with expectations for genome mapping from metagenomics reads, where GC content, repetitive or less well-represented regions often assemble into longer contigs with lower or uneven read depth.^40-42^ These patterns further illustrate the variability of eukaryotic genome recovery from metagenomic stool data, but also confirm that substantial genomic regions can be recovered in high-signal samples (Figure S3B).

**Figure 4.**
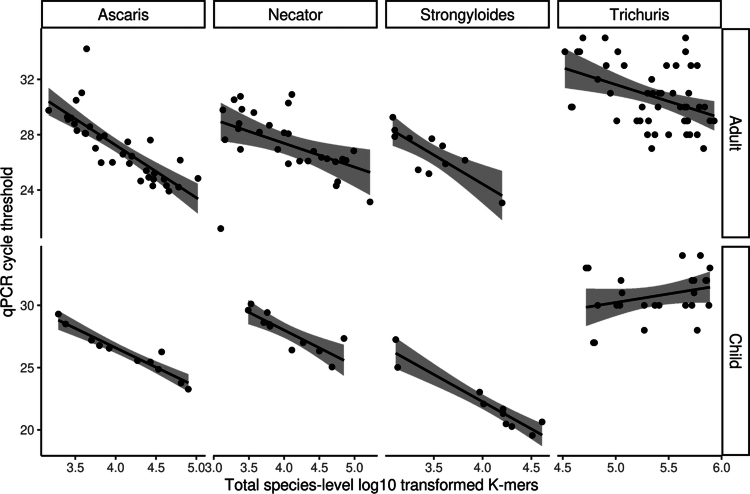
Correlation between metagenome-quantified STH parasite abundances and qPCR. Spearman’s rank correlation between qPCR cycle threshold (Ct) values and log10-transformed species-specific k-mer counts from metagenomic profiling was assessed separately for adults and children. For *Ascaris lumbricoides*, correlations were strong and significant ​​(Adults: *R² =* 0.88*, p =* 2.2 × 10^−12^; Children: *R² =* 0.95*, p <* 2.2 × 10^−16^). For *Necator americanus*, significant correlations were also observed (Adults: *R² =* 0.56*, p =* 0.0013, Children: *R² =* 0.88*, p =* 0.00067). *Strongyloides stercoralis* showed moderate to strong correlations (Adults: *R² =* 0.77*, p =* 0.0056, Children: *R² =* 0.92*, p =* 0.0001). For *Trichuris trichiura*, the correlation was weaker and only significant in adults (Adults: *R² =* 0.37*, p =* 0.008, Children: *R² =* 0.26*, p = 0.21*). Ct values are inversely related to parasite load. Only correlations with p ≤ 0.005 were considered significant.

We next compared metagenomic detection with qPCR. Out of the 310 samples, the k-mer-based method identified 97 positives and 213 negatives, corresponding to a sensitivity of 82,47% and a specificity of 87,32% relative to qPCR (Table S11). The qPCR cycle threshold (Ct)(Figure S3C) values were strongly and significantly negatively correlated with metagenomic k-mer counts for *A. lumbricoides* in both adults (*R²* = 0.88, *p* = 2.2 × 10^−12^) and children (*R²* = 0.95, *p* < 2.2 × 10^−16^), and similar trends were observed for *N. americanus* (adults: *R²* = 0.56, *p* = 0.0013; children: *R²* = 0.88, *p* = 0.00067) and *S. stercoralis* (adults: *R²* = 0.77, *p* = 0.0056; children: *R²* = 0.92, *p* = 0.0001). For *T. trichiura,* correlations were weaker and non-significant in children (*R²* = 0.26, *p* = 0.21) but moderate and significant in adults (*R²* = 0.37, *p* = 0.008) (Figure 4). Although both qPCR and metagenomics sequencing ultimately detect parasite DNA in stool, they do so via different mechanisms, qPCR amplifies species-specific genetic markers, whereas metagenomics classifies sequence fragments within the total read pool. Consequently, differences in detection, particularly for *T. trichiura*, may arise from biological and technical factors, including variable DNA yield due to egg morphology or wall composition, uneven distribution of parasite material in stool, inefficient lysis during DNA extraction, intermittent egg shedding, or stage-specific DNA content. Cross-reactivity of qPCR primers with non-target nematodes could further contribute to discordance between methods. Taken together, these results show that combining k-mer-based classification with genome coverage assessment offers a practical proof-of-concept approach for detecting STH parasites from metagenomic stool data. Performance was consistent for most species, and coverage profiles support the reliability of detection in high-signal samples. Notably, this approach outperformed the reference-based tool EukDetect,^43^ which failed to detect the prevalent *A. lumbricoides*, *T. trichiura*, and *N. americanus* in our dataset (Figure S3D).

### Co-occurrence of the gut microbiome and STH parasites

**Figure 5.**
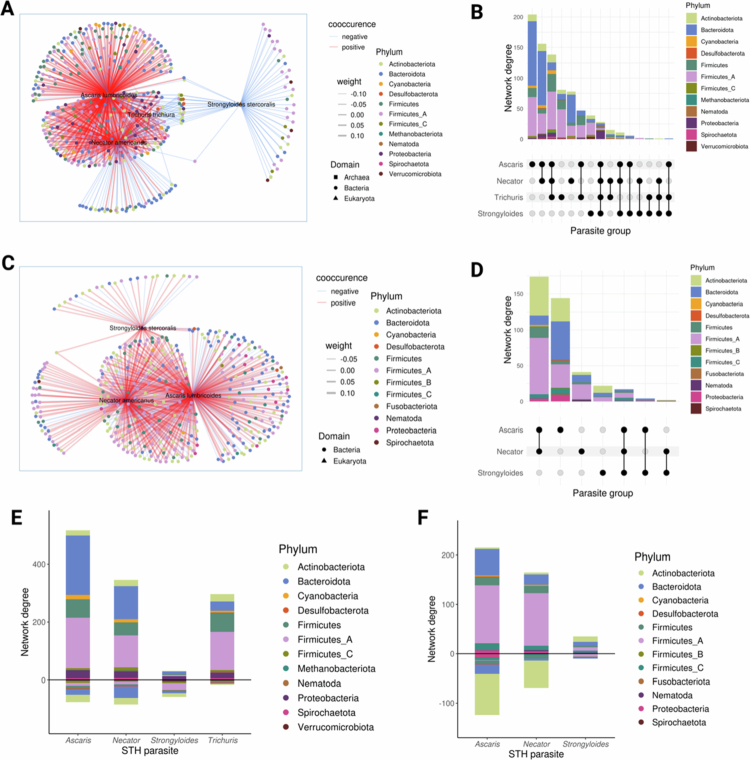
Co-occurrence network plots for adults and children in the Gabon dataset. A) Co-occurrence network for adults, displaying species with significant co-occurrence probabilities. **B)** Network degree (number of connections per node) within the adult co-occurrence network. **C)** Upset plot showing parasites linked to similar bacteria/archaea in the adult network. **D)** Co-occurrence network for children, displaying species with significant co-occurrence probabilities. **E)** Network degree (number of connections per node) within the children's co-occurrence network. **F)** Upset plot showing parasites linked to similar bacteria/archaea in the children's network. Nodes represent species with significant co-occurrence probabilities, with red indicating positive co-occurrence and blue indicating negative co-occurrence. Only species associations with weights between −0.14 and −0.01 (negative) and 0.05 to 0.15 (positive) are shown for visualisation.

Using probabilistic models based on metagenomic data, we inferred co-occurrences between microbes and STH parasites presence/absence. Presence/absence data were used as required by the cooccur R package, which implements a probabilistic model of species co-occurrence based on binary input. In adults, all 4 STH parasites showed co-occurrences with specific microbial species (Figure 5A,B), while in children, only 3 parasites (excluding *T. trichiura*) exhibited such co-occurrences (Figure 5C,D). Consistent with the association between parasite species count and increased microbial diversity, parasite species presence tended to be positively associated with bacterial presence, especially in adults (Figure 5B,E). Ascaris had the highest number of unique co-occurrences with microbes in both adults and children (Figure 5E,F). Positive associations were frequently observed between parasites and Firmicutes or Bacteroidota, while negative associations were observed between parasites and Actinobacteriota (Figure 5E,F). Furthermore, analysis of random co-occurrence graph subsets indicated a low probability of associations between parasites or bacteria with similar properties (Figure S3E). Overall, our findings support the associations of microbial species with parasite richness and suggest that these STH parasites may help shape specific microbial niche compositions.

### Consistent parasite-microbiome associations across African datasets

**Figure 6.**
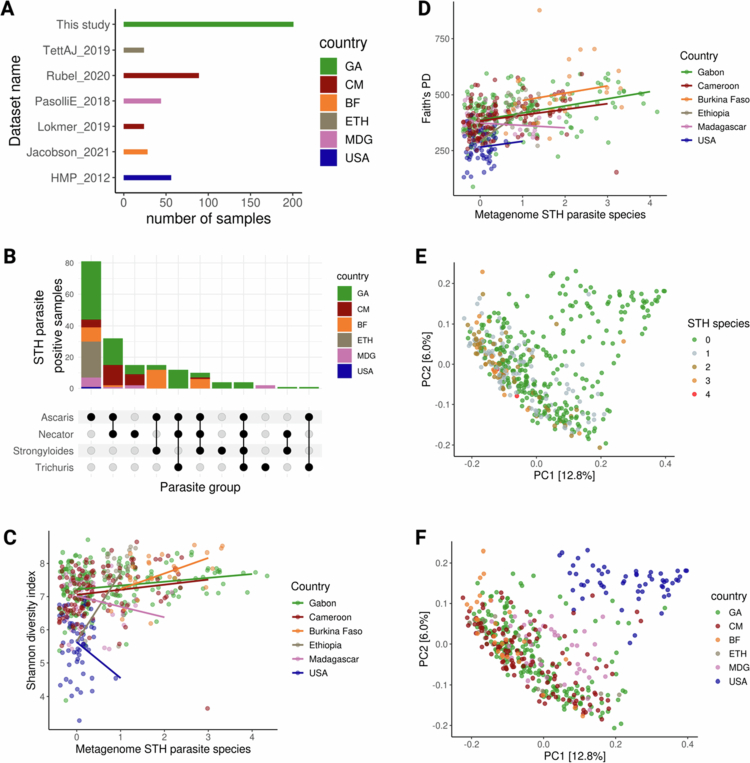
Meta-analysis of soil-transmitted helminth (STH) burden and gut microbiome diversity across multiple African cohorts.A) The distribution of samples based on the STH parasites detected, categorised by datasets (GA = Gabon, CM = Cameroon, ETH = Ethiopia, GHA = Ghana, MDG = Madagascar, TZA = Tanzania, and USA). **B)** Upset plot showing STH parasites detected from metagenomes across datasets, with colours representing the country of origin. **C)** Correlation between Shannon diversity index and the number of STH parasites across datasets, using a generalised linear model (GLM). **D)** Same as panel C, showing correlation between Faith’s PD and the number of STH parasite counts across datasets (GLM). **E)** Principal Coordinate Analysis (PCoA) of unweighted UniFrac values, with samples coloured based on the number of STH parasites detected. **F)** PCoA of unweighted UniFrac values, with samples coloured by country.

We leveraged publicly available metagenomic datasets to assess whether associations between STH parasites and gut microbiome are consistent across African populations. Using parasite species counts inferred from k-mer matches to parasite reference genomes, we conducted a meta-analysis across five metagenome datasets from Gabon (this study), Cameroon,^27^ ^,^ ^44^ Burkina Faso,^45^ Ethiopia,^46^ and Madagascar,^47^ along with an American reference cohort.^48^ Due to limited representation of young children in these metagenomic studies, we focused our analysis on adult women (mean age = 32), aligning with our primary cohort from Gabon. In total, 466 samples were included in the meta-analysis (410 from African cohorts; 405 with Age data; Figure 6A).

We first examined the prevalence of four STHs based on metagenomic detection. *A. lumbricoides* was the most prevalent species, particularly in Burkina Faso (100%) and Ethiopia (95.8%), with lower detection in Gabon (38.3%), Cameroon (17.1%), Madagascar (16.7%), and USA (1.8%). *S. stercoralis* prevalence ranged from 64.3% in Burkina Faso to 0% in several populations, including Ethiopia and the USA. Prevalence differences for all four STHs were statistically significant across populations (*χ²* tests, FDR-adjusted *p* < 0.001; Figure 6B; Figure S3F), with STHs largely undetectable in the American dataset.

Across datasets, we observed that higher parasite species richness was significantly associated with increased microbial alpha diversity. Specifically, Shannon entropy was positively associated with parasite richness (estimate = 0.14, FDR-adjusted *p* = 0.00124; Table S4a), while Faith’s phylogenetic diversity also increased with parasite richness (estimate = 31.5, FDR-adjusted *p* = 2.08 × 10^−10^; Table S4b; Figure 6C,D). In contrast, neither age nor GDP per capita showed significant associations with alpha diversity after FDR correction (all FDR-adjusted *p* > 0.8). These associations remain significant after accounting for between dataset differences (random intercept), which predominantly, but not exclusively, corresponded to sampling country and protocols.

We also observed weak clustering by parasite species richness and dataset in the PCoA analyses (Figure 6E,F). PERMANOVA confirmed that parasite species richness was significantly associated with beta diversity in Burkina Faso, Cameroon, Ethiopia, Gabon, and Madagascar across multiple distance metrics. Effect sizes were modest, generally ranging from *R²* = 0.01–0.07, but reproducible across sites (Table S4C). When pooling all datasets, parasite richness explained a consistent and statistically significant proportion of variance across metrics (*R²* = 0.017–0.020). Age contributed a smaller effect (*R²* = 0.007–0.012), while socioeconomic context (GDP per capita) explained a comparable amount (*R²* = 0.019–0.029). Geography (country) had the strongest effect, ranging from *R²* = 0.035–0.087 depending on the distance metric (Table S4c). These results show that parasite richness makes a reproducible but modest contribution to gut microbiome composition across African cohorts, with effects consistently smaller than those of geography or socioeconomic context.

**Figure 7.**
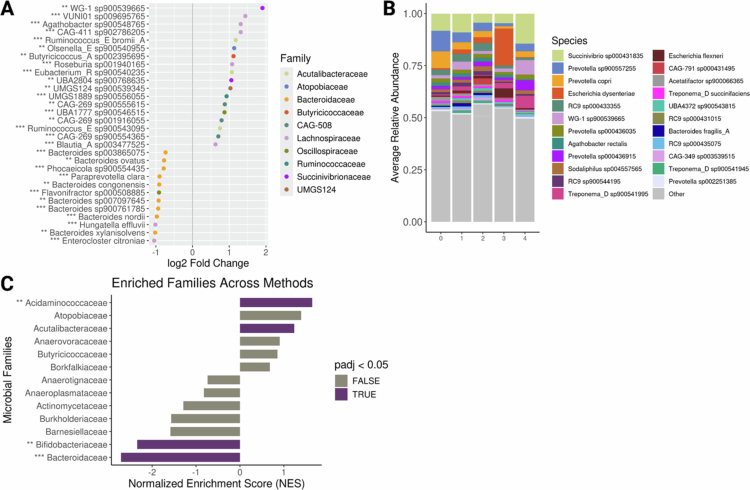
Differential abundance of microbial species by STH parasite burden across countries **A**) Log2 fold changes in microbial species abundance associated with the number of STH (0−4) species based on DESeq2, MaAslin2, and ANCOM-BC. Shown are the top 30 species identified as significant (FDR-adjusted *p* < 0.05), by at least two of the three methods. Species detected by all three methods are marked with ***, and those by two methods with **. Analysis accounted for country as design factor and *p*-values were corrected using the Benjamini-Hochberg method. **B**) Relative abundances of all differentially abundance species by≥2 methods. The top 23 species are coloured by the bacterial family; remaining are grouped as “Other”. **C**) Normalised enrichment scores of bacterial families significantly overrepresented among differentially abundant species, based on fast gene set enrichment analysis (FGSEA).

To identify microbial species consistently associated with parasite richness, we used the intercept of the results from DESeq2, MaAslin2 and ANCOM-BC after filtering for species prevalence (FDR-adjusted *p* < 0.05) and controlling for geography and age. This yielded 665, 1050 and 1110 significantly associated species respectively, with 1072 overlapping across methods (Figure 7A,B, Table S5a).

Using FGSEA, we identified 20 bacterial families overrepresented among the differentially abundant species. These included members of Clostridia (*n* = 5), Bacteroidia (*n* = 5), Gammaproteobacteria (*n* = 2), Coriobacteria (*n* = 2) and one family each from Actinomycetia, Bacilli, Desulfovibrionia, Negativicutes, Spirochaetia and Vampirovibrionia. Most of these showed positive normalised enrichment scores (NES), suggesting an enrichment of taxa positively associated with parasite richness (e.g., all five Clostridia families, two Gammaproteobacteria and others). In contrast, a smaller number of families, including Bacteroidia (*n* = 4) and one each from Actinomycetia and desulfovibrionia, had negative NES values, indicating depletion (FDR-adjusted *p* < 0.05; Figure 7C, Table S5b).

Together, these findings indicate that associations between STH richness and gut microbiome diversity and composition, initially identified in Gabon, are also observed in other African populations using metagenomic data. While these patterns do not imply causality, they suggest that STH infections may be consistently linked with microbial diversity and compositional shifts in diverse African adult populations.

## Discussion

Infection with STH is a neglected tropical disease that exerts complex, context-dependent effects on the human gut ecosystem. In this proof-of-concept study, we integrated molecular detection of STH with shotgun metagenomics to explore the relationship between STH infection and gut microbiota in 155 mother-child pairs from rural and semi-urban Gabonese communities. By combining qPCR assays with k-mer based detection from metagenomic data, we provide insights for the ecological interplay between STH parasites and the human gut microbiome.

One of the key methodological strengths of this study is the dual use of qPCR and shotgun metagenomics for STH detection. While traditional microscopy remains the clinical standard, it is laborious and often impractical in metagenomic studies. Our approach showed good concordance between molecular methods, with qPCR offering high sensitivity and metagenomic detection yielding>80% sensitivity and specificity. Additionally, our validation of shotgun metagenomics against qPCR revealed robust correlations for most STH species. Notably, *T. trichiura* detection remained challenging, reflecting issues with primer specificity, egg shedding dynamics and DNA extraction efficiency. STH prevalence in our Gabonese cohort was high, particularly among mothers, which aligns with prior surveys from Gabon reporting adult STH prevalence>20%,^49^ ^,^ ^50^ with STH infections in children reported between 20 to 33%.^51^ ^,^ ^52^ In Lambaréné, where healthcare access is more established, this high maternal burden relative to children may reflect age-and behaviour-related reinfection dynamics, occupational exposures, or patterns of treatment uptake. In contrast, in the more remote Ikobey region, similarly high infection rates across mother-child pairs suggest persistent community transmission driven by limited sanitation and reduced access to preventive care.

The observed context-specific associations between STH burden and gut microbiome diversity likely reflects differences in environmental exposure, host development, and the stability of the microbial ecosystem across age groups. In rural Ikobey, where parasite exposure is widespread, increased parasite richness may promote or reflect a more heterogeneous microbial environment, particularly in children whose immune systems are still maturing. This may arise from immunomodulatory effects of helminths that enable colonisation by a broader array of microbes, or from shared transmission routes linking parasite and microbial exposure via contaminated environments. In contrast, the absence of clear associations in adults or in the urban setting of Lambaréné suggests that the mature adult microbiome is more resistant to perturbations and that hygiene infrastructure and periodic anthelminthic treatment may buffer parasite-microbiota interactions in this community. The strong influence of age group on microbial structure in Lambaréné, where parasite prevalence is lower, further underscores the importance of developmental transitions, potentially including diet, diversification, or immune maturation, in shaping the microbiota independently of STH exposure. The observed positive associations between parasite richness and microbiome diversity in Ikobey children, aligns with a previous study from Cameroon, where an increase in alpha diversity was associated with the number of parasites, although no differentiation between children and adults was made,^44^and other global reports indicating increased microbial richness in helminth-infected individuals.^53^ ^,^ ^54^ In contrast, a study conducted in Tanzania reported increased alpha diversity in parasite-infected mothers, whereas in children the effect was more limited, with only bacterial richness (Chao 1) increasing while other diversity measures did not show significant differences.^55^ These discrepancies may be attributed to regional differences in parasite species and host responses. Nevertheless, the associations between parasite richness and microbial richness in this study are modest compared to the variance explained by age and location, highlighting that microbial community structure remains largely shaped by local environmental factors. Together, these patterns support a model in which parasite-microbiota associations are modulated by both host age and ecological context.

We observed specific microbial taxa associated with parasite infections. Notably, species from the Bacteroidaceae, Bifidobacteriaceae, and Lachnospiraceae families showed negative associations with parasites, in both locations in Gabon and the meta-analysis dataset. *Bifidobacterium* is a taxonomic group often associated with infant gut microbiota and may reflect developmental differences or microbiome maturity stages, rather than a direct link with parasitism. Conversely, the majority of species with a positive parasite association were Bacteroidales. Previous studies in humans have reported an increased abundance of Bacteroidales in parasite-infected samples,^44^ ^,^ ^56^while mouse models of helminth infection have shown a decrease in Bacteroidales.^20^ These results suggest that Bacteroidales may interact differently with helminth infections across host species, as human studies tend to report increases while mouse models often show decreases. Such apparent contradictions in literature may therefore arise from both biological differences between hosts and from limited taxonomic resolution in earlier analyses. Previous reports have also described an increase in the relative abundance of Clostridiales in the presence of STH parasites, often accompanied by a shift in relative proportions from Bacteroidota to Firmicutes.^55^ ^,^ ^57-59^ However, these findings are based on compositional data, where increase in one group necessarily implies decrease in another, and thus should be interpreted with caution. Additionally, it is important to emphasise that most differential taxa were method- and context-specific, with only a small subset reproducibly detected across locations and methods, highlighting the challenges of robust biomarker identification in microbiome–parasite studies.

It is important to also note that various species within these families associate differently with parasite infections, which may also result from analysis of compositional data. Interestingly, several of the bacterial taxa that correlated positively with parasite species richness in our data and meta-analysis are known mucus degraders.^60^ Studies on mice have demonstrated that the absence of mucin production impairs their resistance to infection by *Trichuris muris*, a common mouse parasite.^61^ It is plausible that when STH parasites colonise the gut, the bacterial mucin degradation dynamics are altered, resulting in shifts within the mucin-associated microbial communities.

In addition to taxonomic insights, functional profiling revealed metabolic associations tied to parasite status. In Lambaréné (low STH prevalence), only minimal functional differences were detected, indicating limited alteration to microbial metabolic capacity. However, in Ikobey (higher prevalence), we identified 27 pathways consistently associated with parasite positivity across the three statistical methods. Most of these pathways, particularly those involved in amino acid biosynthesis (e.g., threonine, methionine, lysine, histidine, valine, arginine) and central carbon metabolism (TCA cycle, pyruvate fermentation, coenzyme A biosynthesis), were depleted in parasite-positive individuals. These patterns echo reports of diminished microbial biosynthetic activity and shifts in energy metabolism during helminth infections in animal studies.^62-64^

Only two pathways were enriched in parasite-positive samples: the purine nucleotide salvage superpathway and GDP-mannose–derived O-antigen biosynthesis. Both play critical roles in microbial adaptation to stress. The purine nucleotide salvage superpathway conserves cellular resources by recycling existing purine nucleotides, such as adenine and guanine, rather than relying on energy-intensive de novo synthesis, a particularly advantageous strategy under nutrient limitation or metabolic stress.^65^ Meanwhile, GDP-mannose–derived O-antigen biosynthesis^66^ is essential for maintaining bacterial cell-envelope integrity. GDP-mannose serves as a key precursor for the synthesis of O-antigen structures that reinforce the outer membrane. In a recent study, disruption of O-antigen biogenesis in *Escherichia coli* was shown to increase susceptibility to bile salts, a physiologically relevant gut stressor, highlighting its role in stress protection.^67^

Depletion of the Bifidobacterium shunt, an obligate fermentative route in *Bifidobacterium* spp. characterised by fructose−6-phosphate phosphoketolase (F6PPK), was also consistently observed to be depleted in parasite-positive individuals in Ikobey. This pathway facilitates efficient carbohydrate fermentation into acetate and lactate, contributing to gut barrier maintenance^68^ and immune modulation.^69^ Importantly, both Bifidobacterium taxa and the shunt pathway were depleted even after adjusting for age group, suggesting that in addition to normal developmental variation parasite infections may also contribute to these changes. Supporting this, in a pig model of *Ascaris suum* infection, administration of *Bifidobacterium animalis* subsp. *lactis* (Bb12) helped mitigate infection-induced impairments in glucose absorption and localised immune responses.^70^ Together, these functional findings align with and extend the taxonomic shifts we observed, demonstrating that higher parasite burden is associated with coordinated metabolic changes in the gut microbiome, particularly reductions in biosynthetic and fermentative capacity and selective enrichment of stress-responsive pathways.

The co-occurrence network analysis provided further insights into the ecological relationships between STH parasites and the gut microbiota. We observed significant associations between specific bacterial species and STH parasites, particularly in adults where all four STH species showed distinct co-occurrences with microbial taxa. Ascaris showed the highest number of unique co-occurrences, often with Firmicutes and Bacteroidota, indicating potential niche preferences and microbial community adaptations in response to parasitic colonisation. These results suggest that STHs do not induce a uniform shift in microbiome composition, highlighting the nuanced nature of parasite-microbiota interactions in the gut ecosystem.

Expanding beyond Gabon, our cross-cohort meta-analysis indicates that helminth infections are associated with reproducible but modest shifts in gut microbial diversity across African populations. Although the effect sizes were small relative to geography and socioeconomic context, the consistent increase in alpha diversity and enrichment of certain Clostridia taxa suggest a consistent relationship between helminth and bacteria within the gut microbiome. One possible interpretation is that common environmental exposures, such as contamination, simultaneously shape parasite prevalence and microbial diversity; alternatively, helminth colonisation itself may create ecological niches that favour specific microbial groups. Experimental work supports that helminth-induced immune modulation and altered microbial metabolism can reshape the gut microbiome. For example, murine infection with *Heligmosomoides polygyrus* increased short-chain fatty acid (SCFA) production and conferred anti-inflammatory effects, changes that were transferred via faecal microbial transplantation and dependent on host GPR41 signalling.^71^ At the same time, the strong influence of geography highlights that helminth–microbiome interactions are embedded within local ecological and cultural contexts, cautioning against broad generalisations across populations.^44^ ^,^ ^72^

Despite the strengths of our study, such as high-depth metagenomic sequencing and robust validation techniques, there are limitations that warrant consideration. The observational and cross-sectional nature of the data limits our analyses to only infer associations. While metagenomic detection methods were carefully validated against qPCR, and read mapping, inherent biases related to DNA extraction, sequencing depth, and reference genome representation remain. Geographic and socioeconomic variation also contributes substantially to microbiome structure, sometimes more than parasites alone, and complicates cross-cohort comparisons. For instance, while parasite-microbiome associations were reproducible across African datasets, they remained modest relative to geographic structuring. This study is further limited by its focus on mothers and young children in only two Gabonese locations, with different lifestyles and parasite prevalence. Additionally, the lack of detailed deworming histories, dietary assessments, and environmental microbiota exposure limits our ability to account for confounding variables. Furthermore, because analyses were stratified by location to account for geographic and demographic heterogeneity, some parasite-microbiome associations were site-specific in direction or magnitude. This context dependence likely reflects genuine ecological variation rather than analytical inconsistency, and emphasises the need for larger, harmonised datasets to disentangle environmental from parasitic effects. Despite these limitations, our findings highlight the value of combining molecular parasite detection with metagenomic microbiome profiling in underrepresented populations. The use of multiple detection platforms and analytical methods bolsters confidence in the robustness of observed patterns.

In summary, our study presents the first assessment of correlations between STH parasite infections and gut microbiota associations in rural communities in Gabon. By including stool samples from villages in a remote community in southern Gabon, we expand the epidemiological understanding of STH and their associations with the microbiome in these populations. Furthermore, our research enhances our comprehension of parasite-microbiome associations across diverse populations in Africa. Overall, our findings underscore the significance of parasite-microbiota associations, particularly in children who are commonly targeted for control interventions through mass drug administration (MDA). According to the most recent WHO data (2022), an estimated 465,348 children in Gabon were in need of preventive chemotherapy against STH infections, including 321,403 school-aged children (SAC) and 143,945 preschool-aged children (Pre-SAC). However, coverage remains suboptimal: only 120,989 SAC (37.6%) received treatment, and just 25.5% of implementation units achieved the effective coverage threshold ( ≥ 75%). National coverage of SAC was only 37.6%,^73^ and the low treatment uptake underscores persistent challenges in MDA programs. One important factor contributing to hesitancy is the high burden of *Loa loa* infection in Gabon, particularly in regions such as Lambaréné, where co-endemicity with malaria, schistosomiasis, and other filarial infections is well documented.^50^ ^,^ ^52^ ^,^ ^74^ Because *Loa loa* microfilaremia increases the risk of severe adverse events, including encephalopathy following albendazole treatment, health authorities and communities often approach deworming campaigns with caution.

Collectively, this proof-of-concept framework demonstrates how integrating metagenomic parasite detection with microbiome profiling across multiple African cohorts can uncover both shared and context-specific patterns of parasite-microbiome interactions. While geographic and demographic variables remain major determinants of gut microbiome structure, our findings establish a methodological and analytical baseline for future comparative studies aiming to disentangle environmental from parasitic influences on microbiome composition in global health context.

Our findings have significant implications for public health strategies, particularly in the design of deworming programs targeting vulnerable populations, such as children in endemic areas. By elucidating the association of STH infections with gut microbiome diversity and community structure, our study highlights the potential role of microbiota modulation in enhancing host resilience against parasitic infections. Further research is needed to explore the mechanistic underpinnings of parasite-microbiota interactions and their implications for host health and disease susceptibility in diverse epidemiological contexts. Future studies would benefit from integrating detailed sociodemographic and lifestyle information, including socioeconomic status, medication use, occupation, housing conditions, to better contextualise observed microbiome-parasite associations.

## Materials and methods

### Study Populations

The study was conducted at the Centre de Recherches Médicales de Lambaréné (CERMEL), Gabon between November 2017 and February 2018. Study participants were mother and child pairs from multiple ethnic groups living in Lambaréné in the vicinity of CERMEL and those living in 5 Ikobey villages including; villages surrounding Tchibanga, Tranquille, Soga, Erouba, and Ikobey of the Southern part of the country (Figure 1A). Given their similar lifestyles, all participants from the 5 villages are grouped as one community and labelled "Ikobey''. Their subsistence practices are centred around hunting, agricultural crop cultivation, and livestock rearing.^75^ ^,^ ^76^ In contrast, participants from and within the vicinity of Lambaréné, a semi-urban town in the Moyen-Ogooue province of Gabon, mostly practice fishing, farming, and a semi-westernised lifestyle. The metadata associated with samples in this study is shown in Table S6.

### Approval and Informed consent

The study was reviewed and approved by the "Comité National d'Ethique '' of Gabon with registration number PROT N˚0025/2017/SG/CNE. Stool samples were collected after participants provided written informed consent or assent.

Sample collection - Stool samples were collected in sterile plastic containers from both mothers and their children. Stool samples collected on-site (CERMEL) were aliquoted and stored at −20 °C within 2 hours of sample collection. Samples collected in the field (Ikobey) were transported in cold boxes to CERMEL and later stored at −20 °C. Samples were later transported to Germany on dry ice where they were stored at −80 °C before DNA extraction, qPCR, and sequencing.

Publicly available datasets - We utilised publicly available African gut metagenome datasets for studies performed in Cameroon,^27^ ^,^ ^44^ Burkina Faso,^45^ Ethiopia,^46^and Madagascar.^47^ The two Cameroon cohorts were sampled from ethnically diverse populations that practiced pastoralist, agropastoralist, or hunter-gatherer lifestyles,^44^or from hunter-gatherers,farmers,or fishing populations in Southwest Cameroon.^27^ The Burkina Faso cohort comprised samples collected from healthy volunteers in a single village in Burkina Faso.^45^ The Madagascar cohort comprised samples collected from two ethnic groups in a remote rainforest region of north-eastern Madagascar^47^ and the Ethiopia cohort included samples from healthy adult females.^46^ We also included one USA cohort, which is a subset of the Human Microbiome Project (HMP) dataset.^48^ Only female subjects who were described as healthy (mostly self-report) and ≥ 17 years old were included. The combined dataset used for our meta-analysis comprised 466 metagenome samples. The metagenomes from the 5 African and one USA cohorts were sequenced on the Illumina platform at an average depth of 45 M read pairs. These metagenomes were retrieved from Sequence Read Archive (SRA) and processed as described here^77^ and here.^44^ The USA cohort was not included in the statistical analysis and was used for illustrative purposes only. The metadata associated with samples in the public datasets is shown in Table S8 and Table S9.

### Detection of parasites by quantitative PCR

Diagnosis of STH parasites was performed by multiplex qPCR using protocols as described elsewhere.^78^ ^,^ ^79^ The target species were *A. lumbricoides*, *T. trichiura*, *S. Stercoralis*, and the hookworm species *N. americanus*. The primers, probes, and reaction conditions for amplification were based on published protocols^79^ ^,^ ^80^ with minor modifications. Primer sequences are given in Table S12. An internal control DNA from Phocine Herpesvirus 1 (PhHV−1) was added into the master mix to control for DNA amplification inhibition. The PCRs were split into two multiplex assays combining *A. lumbricoides, T. trichiura*, and PhHV−1 as targets (assay 1) and *S. stercoralis, N. americanus*, and PhHV−1 as targets (assay 2). The positive controls were plasmids carrying the targeted regions of the four target parasites, kindly provided by the Institute of Tropical Medicine (University of Tübingen). The target genes inserted in these plasmids were an 87 -bp fragment of the ITS1 sequence of *A. lumbricoides*, a 101-bp fragment of the 18S rRNA gene sequences of *S. stercoralis* and *T. trichiura,* and a 101-bp fragment of the ITS2 gene sequence of *N. americanus.* Wells without any template served as negative controls, and all samples, including controls, were tested in duplicate. The total reaction volume was 12.5 μL, which included: 6.25 μL Qiagen HotStarTaq Master Mix (QIAGEN GmbH, Hilden, Germany), 1 μL of 25 mM MgCl_2_, 0.25 μL of 5 mg/ml BSA, 0.1 μL species-specific primers (final concentration of 800 nM), 0.0625 μL of probes (final concentration of 500 nM), 1.5 μL of PhHV−1 plasmid DNA (diluted to 10^−7^), 2 μL of template DNA, 0.2125 μL of PCR-grade water (Sigma-Aldrich, Darmstadt, Germany). The qPCRs were conducted for 40 cycles using a Bio-Rad CFX284 Touch Real-Time PCR Detection System (Bio-Rad laboratories, Germany). Only samples with cycle threshold (Ct) values<36 were considered positive (samples with Ct>35 but<36 were still considered positive). All negative controls had Ct values above 36 (qPCR values are shown in Table S6).

### DNA extraction and shotgun metagenomic sequencing

Total DNA from faecal samples was extracted from approximately 250 mg of frozen stool aliquots. DNA extractions were performed using the Qiagen DNeasy PowerSoil HTP 96 kit (QIAGEN GmbH, Hilden, Germany) following the manufacturer's instructions. Extraction blanks were included to control for reagents and environmental contamination. Extractions were performed under sterile conditions in a laminar flow hood, and the eluted DNA was stored at −20 °C following quantification with Qubit 4 fluorometer (Fisher Scientific GmbH, Schwerte, Germany). Genomic DNA libraries were constructed using the Nextera protocol^81^ with in-house produced Tn5 enzyme.^82^ Briefly, DNA was diluted to 5 ng/µL and tagmented with the Tn5 transposase, followed by amplification using a combination of paired-barcoded primers for 12 cycles. Libraries were pooled and quantified with a Qubit, followed by size selection for the 300−700 bp range on a BluePippin. The resulting libraries were then sequenced on an Illumina HiSeq3000 at the Max Planck Institute for Biology in Tübingen.

### Processing of sequences and taxonomic profiling

All Illumina shotgun raw sequences were processed similarly to Youngblut and colleagues.^77^ Briefly, raw reads were validated with fqtools v.2.0^83^ and de-duplicated with the "clumpify" command from bbtools v37.78 (https://jgi.doe.gov/data-and-tools/bbtools/). Adaptors were trimmed and quality controlled with Skewer v0.2.2,^84^ and the "bbduk" command from bbtools. Human genome reads mapped to the hg19 assembly were filtered with the "bbmap" command from bbtools.

Quality control (QC) reports for all reads were generated with fastqc v0.11.7 (https://github.com/s-andrews/FastQC) and multiQC v.1.5a.^85^ The filtered reads were then subsampled to 5 million reads per sample, and a combination of Kraken 2^86^ and Bracken v2.2^87^ was used to classify filtered, quality-controlled reads with a custom database build from Genome Taxonomy Database (GTDB, Release 202; https://gtdb.ecogenomic.org/). Parasite profiling from metagenomic data was performed using KrakenUniq^39^ with a custom database containing the reference genomes of the four target STH species, *A. lumbricoides*, *S. stercoralis*, *N. americanus*, and *T. trichiura* (accessions in Table S10). Raw reads were first classified with Kraken2 to identify and remove Bacterial and Archaeal sequences; the remaining unclassified reads were then queried against the STH database.^34^ Bacteria and Archaea classification with Kraken2 were resolved to species level. KrakenUniq assignments were resolved at the species level based on unique k-mers. To reduce spurious assignments from low-complexity or contaminant sequences, we applied a minimum detection threshold of 1000 unique k-mers per species per sample, as recommended by the tool’s authors. Counts below this threshold were set to zero (k-mer counts are shown in Table S7). The resulting k-mer counts were used both as abundance estimates and, when binarized, as presence/absence calls. This approach was intended as a proof-of-concept for direct STH detection from shotgun metagenomes, with further validation performed through reference-based mapping and coverage analysis (described below).

### Assessing parasite genome assembly quality and coverage

Reference genomes for all four target STH species were downloaded from NCBI and indexed with bowtie2-build (Bowtie2 v2.3.5).^88^ Metagenomic reads classified as “unclassified” by Kraken2 were mapped to these references using Bowtie2, and per-base coverage was calculated across each contig. Coverage metrics were summarised in R,^89^ including contig length, median coverage, minimum/maximum average converge per contig. BLASTn searches of high-coverage contigs confirmed sequence identity to the respective target parasite species. The overall sample processing workflow is shown in Figure S4.

### Statistical analysis

The mean ages and proportions of STH parasite infections were compared between groups using the Wilcoxon test. The assessment whether STH infection status was more similar within mother–child pairs than expected by chance was performed separately for each sampling location (Ikobey and Lambaréné). For each parasite species (*A. lumbricoides, T. trichiura, N. americanus*, and *S. stercoralis*), as well as for any-parasite positivity, binary infection status was defined using qPCR cycle threshold values<36. For each location, parasite combination, mother–child concordance was calculated as the proportion of pairs where mother and child had the same infection status. The null distribution was generated from 10,000 permutations in which child infection statuses were randomly shuffled across pairs within the same location, preserving the number of positive and negative children. The *P*-value was defined as the proportion of permuted concordance values greater than or equal to the observed concordance.

All diversity measures were obtained from shotgun metagenomic data. Reads were randomly subsampled to 5000000. Diversity was assessed using QIIME 2^90^ at a rarefaction depth of 300000 reads. The alpha diversity measures assessed include Shannon's index (which takes into account the abundances and evenness of species), and Faith's PD (which takes into account the phylogenetic relatedness of species). Beta diversity was assessed using Bray Curtis (takes into account species abundances) and Jaccard (measures presence/absence of species) dissimilarity indices. For the Gabon-only cohort, metadata variables tested with alpha and beta diversity in R include the number of parasites, age group, age z-scores and location. We accounted for potential familial clustering in microbial diversity analyses by including *FamilyID* (linking mother-child pairs within households) as a random effect in linear mixed-effects models for alpha diversity, and as a stratification variable in PERMANOVA for beta diversity. In both cases, however, *FamilyID* explained negligible variance, and in the mixed models this resulted in singular fits. Given this, and consistent with prior work in the same population showing both intra-household and inter-household strain sharing,^31^ we proceeded with linear models that did not include a family-level effect.

Linear models were used to assess the relationships between alpha diversity and parasite species count, stratified by sampling location (Lambaréné and Ikobey), with age (z-scores) included as a covariate. For beta diversity, PERMANOVA was performed in R using the *adonis* function of the vegan package,^91^ on Bray-Curtis, Jaccard, Weighted, and Unweighted UniFrac distance matrices. In stratified analysis, models included parasite species count, age group, and standardised age (z-scores) as fixed effects. In addition, a pooled PERMANOVA model (without stratification) was run to estimate the effect of location on microbiome composition, where parasite species count, age, and location were included as fixed effects. Similar measures of alpha and beta diversity were employed in the meta-analysis. Linear mixed models were used to assess the relationship between the number of parasites and alpha diversity while accounting for non-independence of samples within the same dataset (random intercept: dataset_name). Since most of the datasets mapped uniquely to sampling countries (1.1), this random effect also adjusted for geographic differences in sampling context. The only exception was Cameroon, which included two independent datasets. PERMANOVA was also performed on beta diversity measures with 999 permutations constrained by dataset ID (dataset_name).

Differential abundance analysis was performed using three complementary methods: DESeq2,^35^MaAslin2,^36^and ANCOM-BC.^37^ These analyses were applied to both the Gabon-only dataset and the broader African meta-analysis cohort to identify microbial species associated with parasite species richness. While DESeq2 remains widely used in RNA-seq studies and some microbiome analysis, it can be prone to inflated false positives when applied to compositional microbiome data, such as those derived from 16S rRNA, gene sequencing or metagenomic shotgun sequencing. These data types often exhibit characteristics like high sparsity, zero inflation, and compositional constraints, which DESeq2 was not originally designed to accommodate.^92^ To mitigate such biases, we employed a consensus overlap approach, reporting only taxa consistently detected by multiple methods. This strategy reduces method-specific noise and highlights reproducible associations across differential-abundance tools.^93^ ^,^ ^94^ For DESeq2, unnormalized microbial species counts were used as input, with singleton reads removed to minimise the influence of sequencing errors. Geometric means were calculated before estimating size factors, and apeglm^95^ was used for log fold changes shrinkage. The design formula included parasite species richness and location for the Gabon cohort, and parasite species richness, country, and age for the meta-analysis. In MaAslin2, species counts were log-transformed and normalised using total sum scaling (TSS). Both minimum abundance and minimum prevalence thresholds were set to 0.0. A linear model was used for the analysis, and continuous variables including age and parasite species richness were standardised using z-scores. The same design variables as in DESeq2 were applied. ANCOM-BC was run using the ancombc() function from the ANCOMBC R package. Parameters included a zero cutoff of 0.90, library size cutoff of 1000, and use of structural zeros, bias correction (neg_Ib = TRUE), and conserve = TRUE setting for conservative inference. The significance threshold was set at *α* = 0.05 with Benjamini-Hochberg correction (p_adj_method = “BH”). The formula used for the Gabon-only dataset was “parasite_count + location”, while for the meta-analysis it was “parasite_count + country + Age”. In both cases, raw unnormalized counts (with singleton reads removed) were used as input, consistent with the other two methods.

For all three methods, microbial species were filtered for prevalence (>50% of samples) prior to analysis, with this step performed separately for Gabon and meta-analysis datasets. Multiple testing correction was performed using Benjamini-Hochberg method, and FDR-adjusted *p*-values below 0.05 were considered significant. Entries with missing values (NA), were removed prior to downstream analyses. To assess whether differentially abundant microbial taxa were enriched at higher taxonomic levels, we applied gene set enrichment analysis GSEA,^38^using the R package *fgsea.* ^96^ All species evaluated in each method (not only those passing the significant threshold) were grouped by family, and their corresponding log2 fold changes were used to rank species. The minimum number of species required per family was set to 2, while the maximum number of species per family was set to 600 for the Gabon dataset and 800 for the meta-analysis dataset, reflecting the large number of DA species identified in the latter. To improve accuracy in *p*-value estimation, the eps parameter in FGSEA was set to zero.

Differential pathway abundance was assessed using HUMAnN3-derived, unnormalized, unstratified pathway abundance profiles. We applied the same three statistical frameworks used for taxonomic differential abundance, DESeq2, MaAsLin2, and ANCOM-BC, stratified by sampling location (Lambaréné and Ikobey). Parasite species count and age group were included as fixed effects. Pathways were considered differentially abundant if they passed an FDR-adjusted *p*-value threshold of 0.1. In general, only pathways identified by at least two of the three methods were considered robust for interpretation. For the HUMAnN3 functional analysis stratified by location, this approach was applied as follows: in the Lambaréné cohort, DESeq2 and MaAsLin2 identified differentially abundant pathways with no overlap between the two methods, while ANCOM-BC did not detect any significant features; in this case, the pathways detected by DESeq2 and MaAsLin2 were reported individually. In the Ikobey cohort, all three methods identified overlapping pathways, and only the intersecting results were reported. This strategy ensures clarity, while emphasising reproducible functional signals and mitigating method-specific false positives. For visualisation, log_2_ fold change estimates were plotted against pathway identity, with overlapping pathways highlighted with stars (*** when detected by all 3 methods and ** when detected by 2 methods).

Co-occurrence abundance analysis was performed with the R package cooccur.^97^ First, the microbiota species abundance counts were split between adults and children. Species counts were then transformed to relative abundances, filtered to a>50% prevalence, and then converted to presence/absence (1/0) in separate datasets. Similarly, the number of parasites from KrakenUniq was converted to presence/absence and then joined to the microbiota presence/absence table in the separate datasets. Probabilities of co-occurrence between microbiota and STH parasites were defined as negative if p_lt<0.05, or positive if p_gt<0.05, where p_lt and p_gt are probabilities that two species do not co-occur less or more frequently than expected. Only the species with significant probabilities of cooccurrences with the 4 STH parasites were included in the network graphs. The weights are the differences between the observed and expected frequencies of co-occurrence normalised by the sample size (*n* = 155) for adults and children respectively.

### Summary of statistical thresholds and multiple testing correction

Unless otherwise specified, all tests were two-sided. Multiple testing correction using the Benjamini-Hochberg false discovery rate (FDR) procedure was applied to analyses involving multiple comparisons, including alpha and beta diversity models (both within the Gabon cohort and in the meta-analysis), chi-square tests of parasite prevalence across sampling locations and countries, and all differential abundance and pathway analyses (DESeq2, MaAsLin2, and ANCOM-BC). FDR-adjusted *p*-values < 0.05 were considered significant, except for pathway-level analyses where a threshold of 0.1 was used. For analyses involving a limited number of comparisons, such as tests for mother-child concordance, linear and logistic models of parasite richness or presence/absence, and comparisons of age or infection prevalence between sampling locations, unadjusted *p*-values are reported.

The following R^89^ packages were used for data processing and visualisations: phyloseq,^98^ microbiome,^99^ dplyr,^100^ tidytable,^101^ tidygraph,^102^ broom,^103^ igraph,^104^ ggplot2,^105^ ggraph,^106^ UpsetR,^107^ complexUpset,^108^ fgsea,^96^ MaAsLin2,^36^ concur.^97^

## Supplementary Material

Supplementary Material KGMI-2587966-supplementary-Figures.

Supplementary Material Supplementary_tables_S1_S12.

## Data Availability

The authors confirm that all data supporting the findings of this study are included within the article and its supplementary materials. The raw sequence data for the Gabon cohort can be accessed from the European Nucleotide Archive under the study accession numbers PRJEB46788 and PRJEB86954. Additionally, the meta-analysis dataset and references are provided in the supplementary tables.
