# Supplementary material for: Infection with gut parasites correlates with gut microbiome diversity across human populations in Africa
Source: Gut Microbes. 2025 Dec 8;17(1):2587966. doi: 10.1080/19490976.2025.2587966 (PMC12688249; doi:10.1080/19490976.2025.2587966)
Supplement: Supplementary Material — KGMI-2587966-supplementary-Figures. [file KGMI_A_2587966_SM5258.docx]

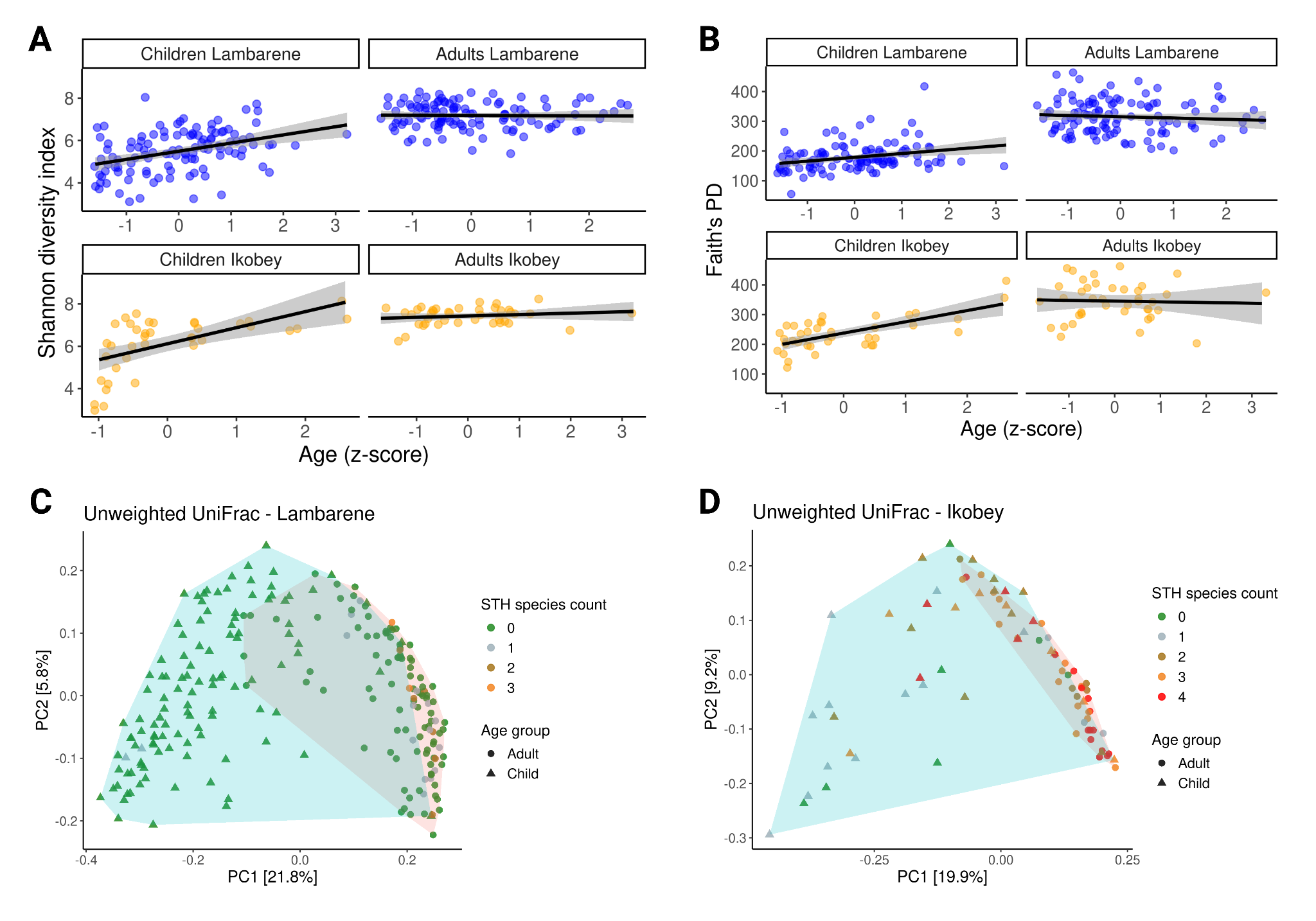


**Figure S1**: **Gut Microbiome Diversity by Age Group and Location**

**A)** Correlation between gut microbiome Shannon diversity index and age z-scores for adults and children by location (correlation method: LM). For children, *R^2^* = 0.33 in Ikobey and *R^2^* = 0.15 in Lambaréné; for adults, *R^2^* = 0.02 in both Ikobey and *R^2^* = 0 in Lambaréné.

**B)** Correlation between Faith’s Phylogenetic Diversity (PD) and age z-scores for adults and children by location (correlation method: LM). For children, *R^2^* = 045 in Ikobey and *R^2^* = 0.08 in Lambaréné; for adults, *R^2^* = 0 in both Lambaréné and Ikobey.

**C)** Principal Coordinates Analysis (PCoA) of unweighted UniFrac values for Lambaréné samples (n = 230), colored by STH species count and shaped by age group (Adults = circles, children = triangles).

**D)** PCoA of unweighted UniFrac values for Ikobey samples (n = 80), colored by STH species count and shaped by age group (Adults = circles, children = triangles).


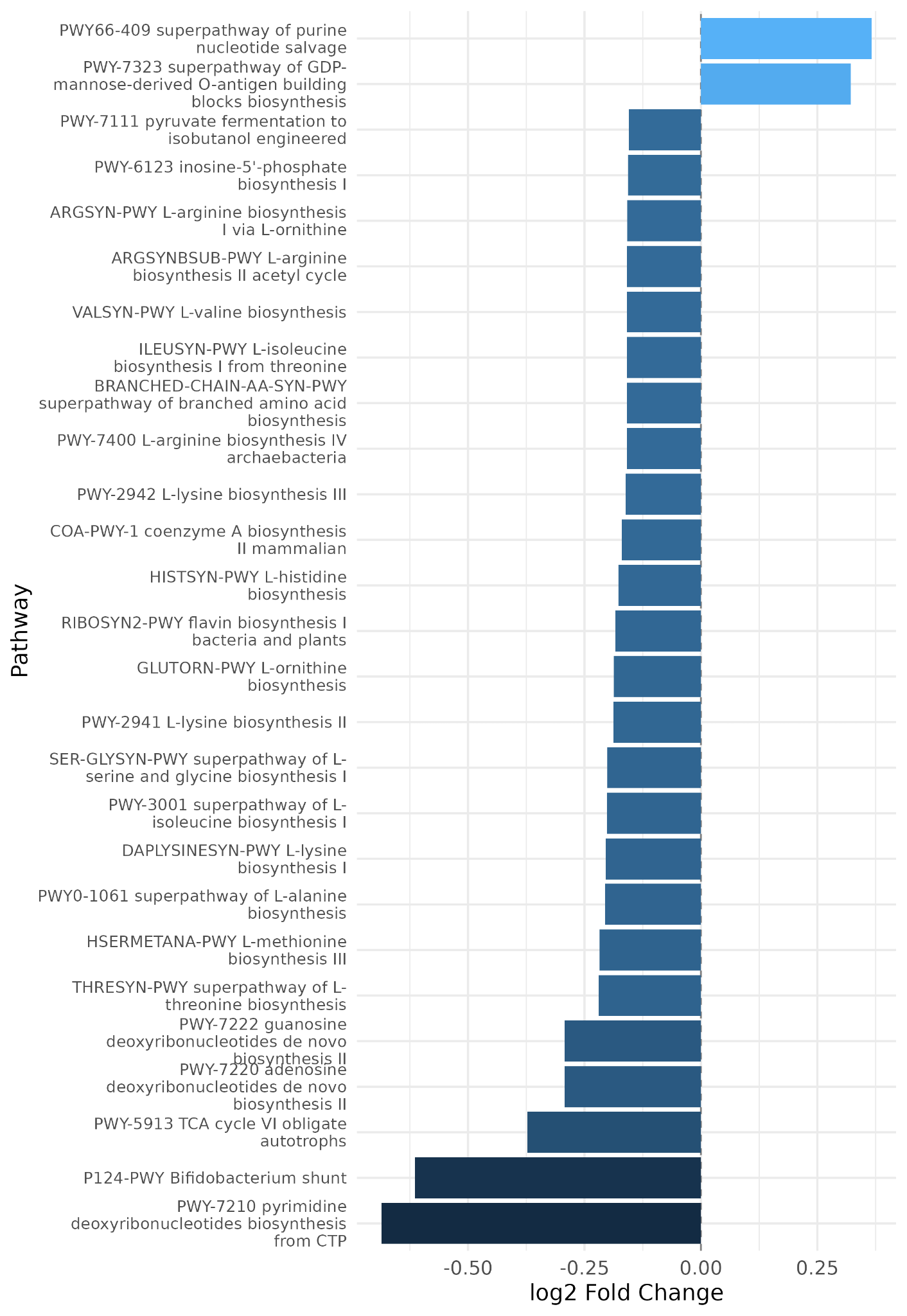


**Figure S2: Differentially abundant pathways in Ikobey**

Log₂ fold change estimates for the 27 pathways identified by all three methods (DESeq2, MaAsLin2, ANCOM-BC). Pathways enriched in parasite-positive individuals are shown with positive values; depleted pathways have negative values. Pathway IDs correspond to MetaCyc identifiers.


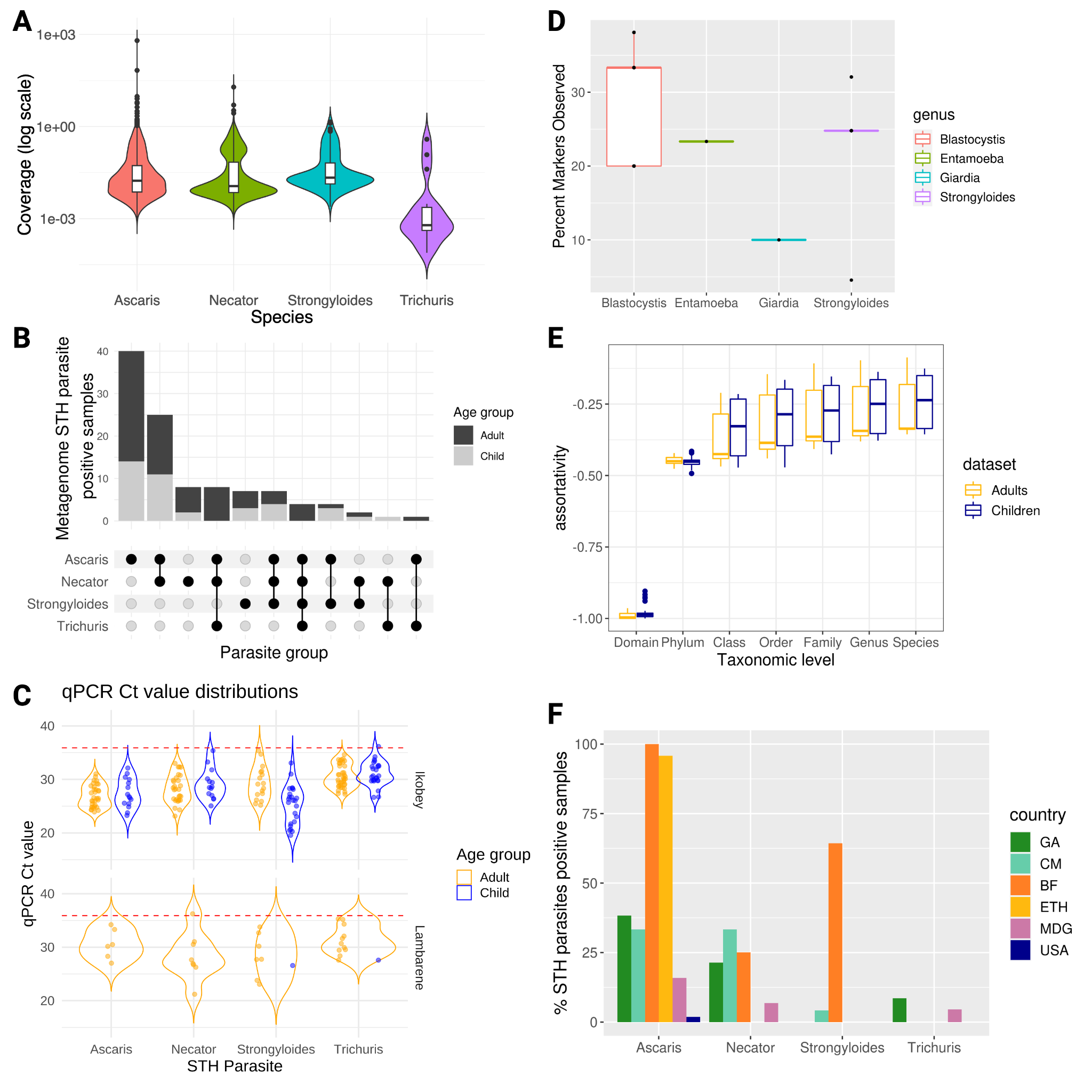


**Figure S3: Detection and characterization of soil-transmitted helminths (STHs) in African gut metagenomes.**

**A**) Boxplots showing the distribution of average coverage depth (log₁₀ scale) for contigs from the reference genomes of *A. lumbricoides*, *N. americanus*, *S. stercoralis* and *T. trichiura*, based on mapping Kraken2-unclassified reads with Bowtie2. Each point represents a single contig; contigs without mapped reads were excluded.

**B**) Upset plots showing co-detection patterns of the four STH parasites in metagenomes from Gabonese mother-child pairs.

**C**) Distribution of qPCR cycle thresholds (Ct) values for the four STH species in samples from Lambaréné and Ikobey. Points represent individual positive samples; violin plots indicate density of Ct values. Lower Ct values reflect higher parasite DNA abundance. The dashed red line marks the positivity threshold.

**D**) Percentage of STH-associated markers detected in Gabonese mother-child metagenomes using the Eukdetect database.

**E**) Boxplots of assortativity coefficients (node connections) across taxonomic levels in co-occurrence networks. For each network, 100 random subsets of 230 nodes were analyzed, and assortativity values calculated per taxonomy level.

**F**) Bar plots showing the percentage of STH-positive samples detected from metagenomes across different datasets, colored by country of origin.


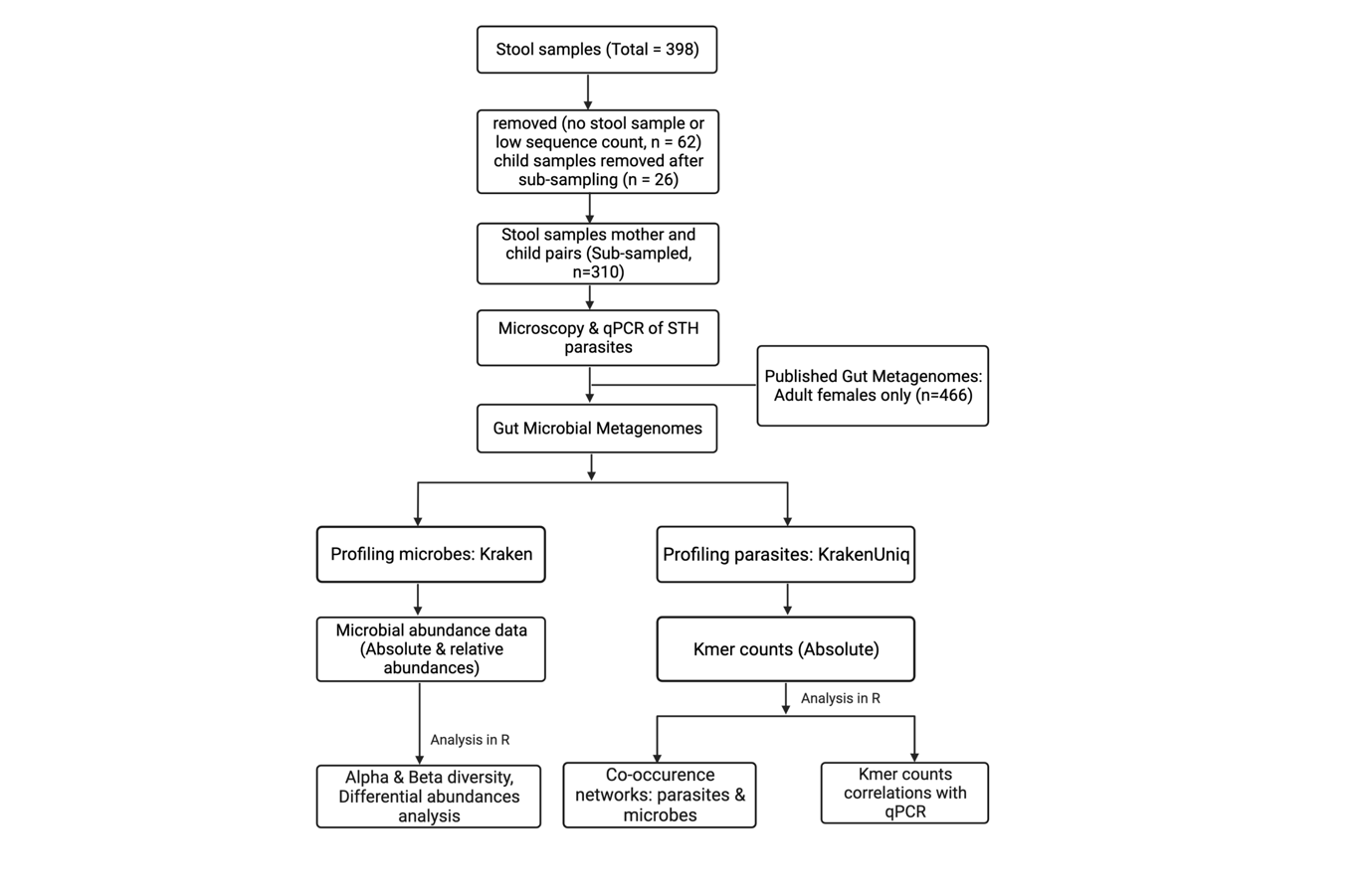
**Figure S4**: **Sample Processing Workflow**

Participants without stool samples and samples with low sequencing counts (<100,000 reads) were excluded from downstream analysis (n = 62). An additional 26 samples were removed to focus on mother-child pairs. Publicly available metagenomes from adult women in studies conducted across four African countries (n = 265) and this study's cohort (n = 201) were included in the meta-analysis. Microbial abundance was estimated using Kraken, and KrakenUniq was used to estimate the number of unique k-mers assigned to each STH parasite.
